# Post-stroke rehabilitative mechanisms in individualized fatigue level–controlled treadmill training in rats

**DOI:** 10.4103/NRR.NRR-D-25-00123

**Published:** 2026-02-05

**Authors:** Yuchen Xu, Yulong Peng, Yuanfa Yao, Xiaoman Fan, Minmin Wang, Feng Gao, Mohamad Sawan, Shaomin Zhang, Xiaoling Hu

**Affiliations:** 1 CenBRAIN Neurotech Center of Excellence, School of Engineering, Westlake University, Hangzhou, Zhejiang Province, China; 2 Key Laboratory of Biomedical Engineering of Ministry of Education, Qiushi Academy for Advanced Studies, Zhejiang University, Hangzhou, Zhejiang Province, China; 3 The Affiliated Huizhou Hospital, Guangzhou Medical University, Guangzhou, Guangdong Province, China; 4 Westlake Institute for Optoelectronics, Westlake University, Hangzhou, Zhejiang Province, China; 5 Department of Neurology, The Second Affiliated Hospital, School of Medicine, Zhejiang University, Hangzhou, Zhejiang Province, China; 6 Department of Biomedical Engineering, The Hong Kong Polytechnic University, Kowloon, Hong Kong Special Administrative Region, China

**Keywords:** central fatigue, corticomuscular coherence, electroencephalography, fatigue, individualized training, interhemispheric balance, motor function recovery, peripheral fatigue, stroke rehabilitation, treadmill training

## Abstract

Clinically, individualized training improves post-stroke motor function rehabilitation efficiency. However, the mechanisms underlying how individualized training facilitates recovery remain relatively unclear. Here, we explored the cortical and corticomuscular rehabilitative effects of post-stroke motor function recovery during individualized training using a rat model of intracerebral hemorrhage. Forced training or individualized fatigue-controlled training was provided from days 2 to 14 post-stroke. The fatigue-controlled training group exhibited superior motor function recovery and less central fatigue compared with the forced training group. Electroencephalograph power spectrum density slope analysis demonstrated better inter-hemispheric balance in the fatigue-controlled training group than in the forced training group. Directed corticomuscular coherence analysis indicated that training-induced fatigue led to a short-term downregulation of descending directed corticomuscular coherence and an upregulation of ascending directed corticomuscular coherence. In the long term, excessive fatigue hindered the recovery of descending control in the affected hemisphere. In conclusion, the individualized strategy of peripheral fatigue-controlled training achieved better motor function recovery, which may be attributed to the mitigation of central fatigue, optimization of inter-hemispheric balance, and enhancement of descending control in the affected hemisphere. This is the first study to investigate the mechanisms underlying individualized rehabilitative effects at the cortical and corticomuscular levels. Our findings suggest that personalized training protocols that are tailored to manage fatigue levels may substantially enhance post-stroke motor efficiency. They also provide a mechanistic foundation for developing fatigue-monitored, individualized rehabilitation programs in clinical practice.

## Graphical abstract

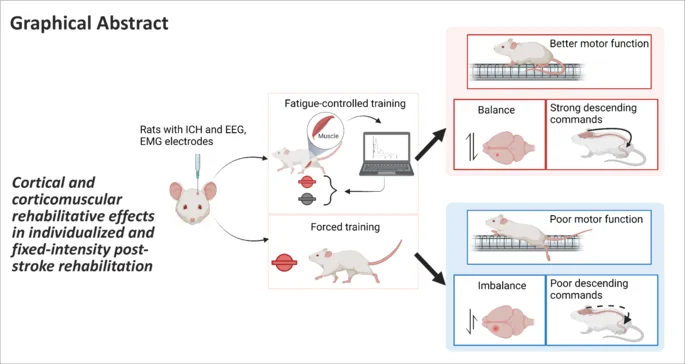

## Introduction

Stroke is the leading cause of chronic disability in adults, with > 50% of survivors experiencing moderate to severe motor function deficits even after routine rehabilitation (Dimyan and Cohen, 2011; Feigin et al., 2021). Early rehabilitation interventions are widely recognized to enhance recovery outcomes (Wang et al., 2022; Wei et al., 2024). However, excessive fixed-training strategies during the early stages can lead to fatigue, stress, and the release of inflammatory cytokines, which may exacerbate secondary brain damage and hinder recovery (Sun et al., 2014; Coleman et al., 2017; Langhorne et al., 2017; Li et al., 2017). In clinical studies, the use of individualized training intensity had shown benefits in post-stroke motor function recovery, especially in patients with initially poor motor function (Al-Jarrah et al., 2014; Miller et al., 2021). Individualized rehabilitation—particularly, those incorporating the modulation of each individual’s fatigue level through heart rate muscle electromyography (EMG)—offer a promising approach to optimizing training intensity during the early stages of rehabilitation. Individualized fatigue modulation is critical because the dual roles of fatigue, as both a promoter of plasticity with moderate fatigue level and a limiter of performance with overloaded fatigue level, mean that it is a key determinant of rehabilitation efficiency (Amann et al., 2013; Kuppuswamy et al., 2015; Li, 2022; Smith et al., 2024). Compared with conventional fixed-intensity training, individualized fatigue modulation strategies have been associated with enhanced neuronal plasticity, improved motor function recovery, reduced stress, and decreased infarct volume (Sun et al., 2014; Chen et al., 2019). Despite these advancements, a critical gap remains in understanding how fatigue-constrained, individualized training intensity provides superior benefits for motor function recovery compared with fixed-intensity training that induces excessive fatigue. Furthermore, there is limited understanding of how individualized training might improve rehabilitation efficiency in different parts of the motor system; this knowledge is essential for optimizing rehabilitation outcomes.

Fatigue is a critical biomarker for evaluating the suitability of rehabilitation training intensity. Overloaded training–induced fatigue hinders functional recovery, whereas low-intensity training without fatigue often yields poor recovery outcomes (Ballester et al., 2022; Mahrukh et al., 2023; Joshi and Dando, 2024; Zeng et al., 2024). Training-induced fatigue occurs in both the central nervous system (central fatigue) and peripheral nervous system (peripheral fatigue). Central fatigue is defined as a reduction in the voluntary activation of muscles, and is often associated with a decreased frequency and synchronization of motor neuron activity and diminished drive from the motor cortex (Tornero-Aguilera et al., 2022). By contrast, peripheral fatigue involves a decline in the contractile strength of muscle fibers, and is often manifested through reduced muscle fiber conduction velocity. Both central and peripheral fatigue are regulated by dynamic negative feedback activity to maintain homeostasis. Peripheral fatigue triggers inhibitory feedback from group III/IV muscle afferents, which then suppress neural command output, thus contributing to central fatigue and mitigating further peripheral fatigue (Amann et al., 2013). Central fatigue reduces motor cortex excitability (McKay et al., 1995; Deshpande and Hu, 2011; Tankisi et al., 2024) and is associated with increased serotonin (or 5-hydroxytryptamine [5-HT]) levels, decreased dopamine concentrations (Meeusen et al., 2006), and potentially impaired cortical neuroplasticity, which is essential for post-stroke rehabilitation (Persico et al., 2001; Wang et al., 2020a). Although peripheral fatigue is frequently used as a metric for rehabilitation monitoring, the evaluation of central fatigue in stroke rehabilitation remains scarce. It is unclear whether rehabilitation strategies that target peripheral fatigue are effective in mitigating the development of central fatigue, thereby underscoring the need for further research in this area.

Post-stroke movement-related rehabilitative effects are observed at both the cortical and corticomuscular levels. At the cortical level, stroke disrupts the interhemispheric balance, leading to increased power in lower frequency bands (i.e., delta, theta) and decreased power in higher frequency bands (i.e., alpha, beta) in the affected (AFF) hemisphere (Finnigan et al., 2016; Cassidy et al., 2020). During rehabilitation, both the restoration of interhemispheric balance and shifts in power bands in the AFF hemisphere have been documented; these changes are considered to be biomarkers of motor recovery capacity (Lefaucheur et al., 2020; Casula et al., 2021). At the corticomuscular level, functional connectivity between the cortex and muscle in descending and ascending pathways during movement execution can be elucidated through directed corticomuscular coherence (dCMC) (Liu et al., 2019). The upregulation of descending dCMC has been reported as the main mechanism underlying motor function recovery (Khademi et al., 2022) and an increase in ascending dCMC to compensate for a reduction in descending dCMC has also been reported (Zhou et al., 2021). These findings underscore the necessity of evaluating rehabilitative effects at both the cortical and corticomuscular levels to better understand the functional mechanisms underlying various training strategies.

The present study addresses these knowledge gaps by investigating how peripheral fatigue–based individualized training interacts with central fatigue and induces rehabilitative effects at cortical and corticomuscular levels to achieve motor function recovery. Our previous study found both FOR-T and FAT-C rehabilitation would benefit the post-stroke motor function compared to no-exercise control stroke group, and FAT-C training benefits more compared to FOR-T group (Xu et al., 2020), whereas it is still not clear about the mechanisms of individualized training. Unlike prior studies that have primarily focused on peripheral fatigue or general outcomes of individualized training, our research uniquely integrated central fatigue evaluation and examined its interplay with peripheral fatigue under tailored training protocols. Additionally, by assessing cortical interhemispheric balance and corticomuscular coherence, this study provided novel insights into the neural mechanisms driving the superior efficacy of individualized training. Such exploration is anticipated to contribute to a deeper understanding of the advantages associated with individualized rehabilitation training and to inform the development of more effective, personalized rehabilitation strategies for stroke survivors.

## Methods

### Animals

Prior evidence indicates that individualized-intensity training is superior to fixed-intensity training for motor recovery (Sun et al., 2014). Our previous study similarly found that both FOR-T and FAT-C improved post-stroke motor function versus a no-exercise control, with FAT-C conferring greater benefit (Xu et al., 2020). To delineate the neural and muscular correlates of these differences, we analyzed electroencephalography (EEG) and electromyography (EMG) during treadmill training to compare FAT-C with FOR-T, omitting a sham stroke model. Forty-two adult male Sprague Dawley rats weighing between 270–310 g, aged 9–10 weeks (purchased from Shanghai SLAC Laboratory Animal Co., Ltd, License No. SCXK (Hu) 2022-004, specific-pathogen-free grade), were included in the study. They were housed one per cage in a vivarium under controlled conditions with a temperature of 22 ± 2°C, humidity of 50% ± 10%, and a 12/12-hour light/dark cycle. The rats had *ad libitum* access to food and water except during experimental periods. Isoflurane was applied for anesthesia induction and maintenance during surgical procedures. The rats with intracerebral hemorrhage (ICH) were randomly divided into two groups: the forced training (FOR-T, *n* = 21) and fatigue-controlled training (FAT-C, *n* = 21) groups. Rats in the two groups underwent different rehabilitation interventions from the subacute to early chronic periods. During rehabilitation, the central fatigue level and the rehabilitative effects at cortical and corticomuscular levels were investigated and compared between the two groups to elucidate how individualized FAT-C contributes to motor function recovery in relation to fixed intensity training. All animal procedures and protocols were approved by the Animal Care Committee of Zhejiang University (protocol number ZJU20210196, 11/8/2021), used the “3R principle” (replacement, reduction, and refinement), and were conducted in accordance with the National Institutes of Health Guide for the Care and Use of Laboratory Animals (8^th^ ed, National Research Council, 2011).

### Training interventions

**Figure 1:**
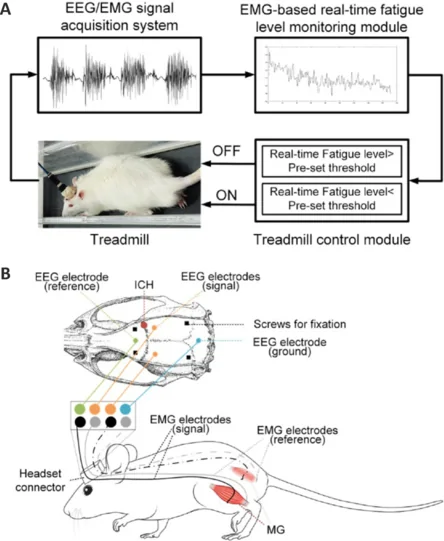
Illustrations of the fatigue-controlled training strategy and surgeries. (A) Illustration of the fatigue-controlled treadmill training platform. The rat ran on the treadmill, and its electromyography (EMG) signal was acquired and amplified by an electroencephalography (EEG)/EMG signal acquisition system and transmitted to the EMG-based real-time fatigue level monitoring module. The fatigue level controlled the on/off state of the treadmill. (B) Illustration of the intracerebral hemorrhage (ICH), the EEG electrodes, the headset connector and the EMG electrodes. The dorsal skull surface of the rat (top) shows the locations of the injection hole (red dots) that induced ICH, the EEG electrodes for EEG signal acquisition in the motor cortex (orange dots), the EEG electrode for the reference (green dots), the common ground (blue dots), and the screws for fixation (black squares). The rat model (bottom) illustrates intramuscular electrode implantation on the medial gastrocnemius muscles. The black and gray solid/dashed lines in the rat model represent the EMG electrodes of the EMG signal (the belly of the medial gastrocnemius muscles) and the EMG reference (tendon), respectively.


FAT-C and FOR-T training were administered as individualized fatigue-controlled and fixed intensity training using a custom treadmill training platform (Xu et al., 2020). Rats in both training modes received treadmill training at a speed of 16 m/min, and accumulated 30 minutes of running per day. The strategic difference lay in whether the 30-minute training session was continuous or intermittently interrupted with rest periods (**Figure 1A**). In the FOR-T mode, rats were compelled to continuously run on the treadmill without rest throughout a single trial. Conversely, in the FAT-C mode, the peripheral fatigue level of the AFF hindlimb was regulated by pausing the training for rest (3 minutes) when the fatigue level exceeded a preset threshold, and restarting it when the fatigue level dropped below a preset threshold. The fatigue level was monitored using the drop rate of EMG mean power frequency (MPF). Briefly,

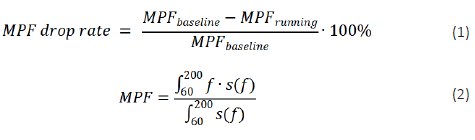


where *s*(*f*) was the power spectrum density of the EMG signal. The MPF value was calculated every 4 seconds and the preset MPF drop rate threshold was 11% (Xu et al., 2020). Because previous studies have reported that moderate training intensity benefits motor function recovery more than low or high training intensity (Sun et al., 2014), and we have demonstrated that the mean maximum MPF drop rate for healthy rats is around 22%, we chose 11% as the moderate fatigue level threshold (Xu et al., 2020). The running MPF was the MPF value, which was calculated based on the EMG of the target hindlimb during treadmill training.

### Animal preparation and surgeries

An accommodation training phase was conducted over 3 consecutive days to select rats who were capable of adapting to continuous treadmill running for the following experiments. The subsequent study required rats to run on a treadmill as a rehabilitation intervention, and three rats failed the accommodation training and were excluded from this study. Thirty-nine rats successfully completed the adaptation training and subsequently underwent surgeries for ICH and the implantation of EEG and EMG electrodes.

The surgeries for ICH induction and EMG and EEG electrode implantation were performed sequentially in a single operation. First, the ICH and EMG electrode implantation surgeries were delivered as described previously (Xu et al., 2020). Briefly, ICH was induced by the stereotaxic injection of type IV collagenase (1.2 µL, 0.25 U in 1 µL NaCl 0.9%; C5138, Sigma, St. Louis, MO, USA) into the caudate putamen (anteroposterior (AP): +0.2 mm, mediolateral (ML): +3.0 mm, dorsoventral (DV): +6.0 mm relative to the Bregma) according to the Waxholm Space Atlas of the Sprague Dawley Rat Brain (Kleven et al., 2023). For EMG, intramuscular electrodes were implanted in the medial gastrocnemius muscles of the AFF and unaffected (UN) hindlimbs using a differential electrode configuration (Teflon-coated stainless-steel wires; AS632, Cooner Wire, Chatsworth, CA, USA) Subsequently, EEG electrodes were implanted. Four stainless steel screw electrodes (diameter: 0.8 mm, length: 1 mm) were threaded into pre-drilled holes in the skull to contact the dura. Each screw electrode was tethered to a silver wire and connected to an eight-pin head connector (**Figure 1B**). The electrodes were assigned as follows: 1) motor cortex signal acquisition (**Figure 1B**, orange dots: AP: –1.75 mm, ML: ±2.25 mm relative to the Bregma), 2) reference electrode (**Figure 1B**, green dot: AP: +2 mm relative to the Bregma), 3) common ground for both EEG and EMG acquisition (**Figure 1B**, blue dot: AP: –2 mm relative to the Lambda). Additionally, four stainless steel screws (diameter: 1.0 mm, length: 1.2 mm) were inserted into the skull periphery to secure the head connector (locations: AP: +2.0 mm, ML: ±2.0 mm to the Bregma; AP: +2.0 mm, ML: ±3.0 mm to the Lambda). The connector was fixed to these screws with dental cement. After suturing the wounds, the rats were placed in a warm recovery box to maintain their body temperature at 36–37°C. Food and water were provided *ad libitum* until the rats fully regained consciousness.

### Randomization

**Figure 2:**
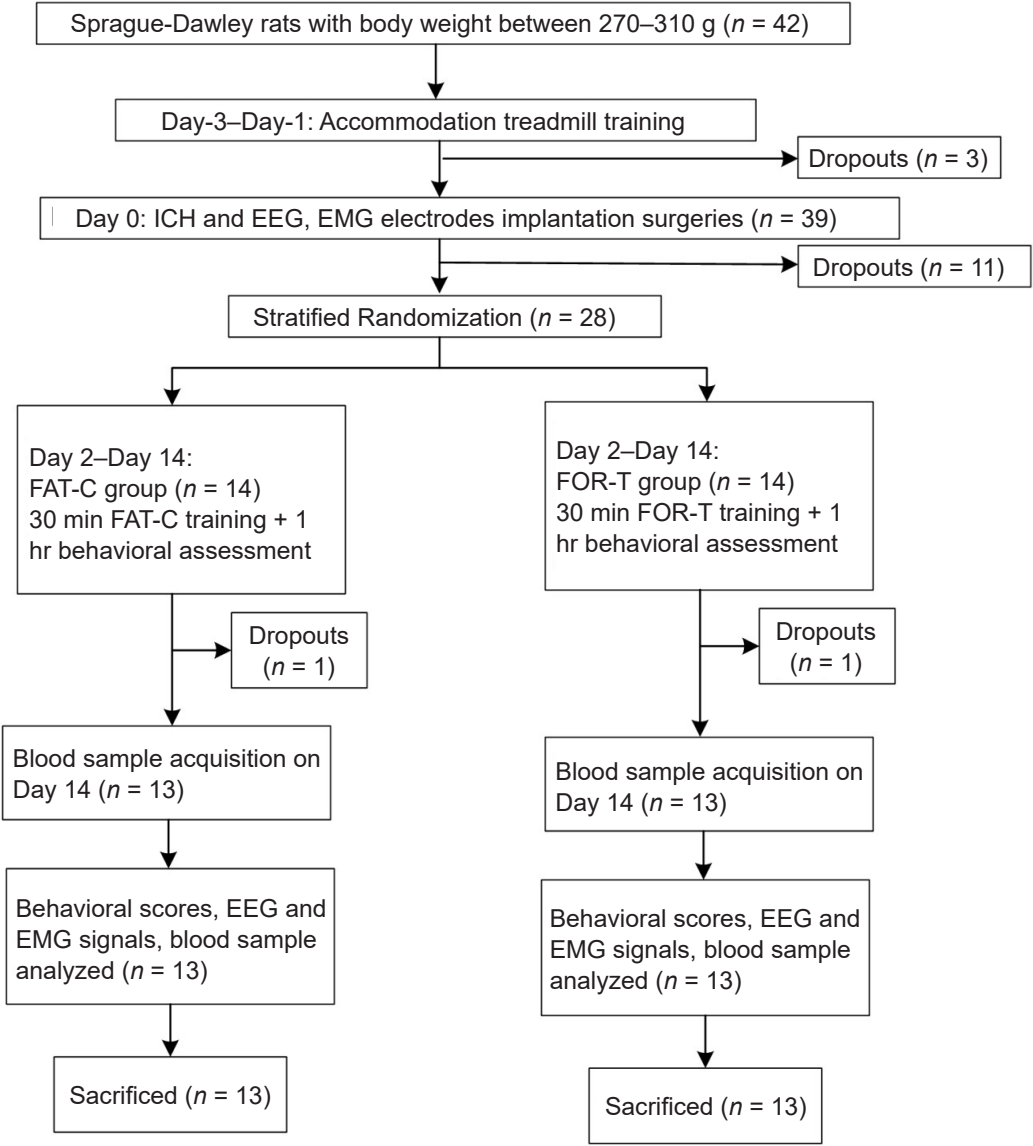
Flowchart of the experimental design. All rats were subjected to three-day treadmill accommodation running followed by intracerebral hemorrhage (ICH), electromyography (EMG) and electroencephalography (EEG) electrode implantation surgeries. Two days after these operations, the rats were randomly distributed into two groups and received forced training or fatigue-controlled training from day 2 to day 14 poststroke. Changes in central fatigue, interhemispheric balance, and the corticomuscular pathway were evaluated to reveal the mechanisms of individualized peripheral fatigue-based training.

After a 2-day recovery period following the surgeries, 11 rats were excluded because of mild/no motor function deficits or motor function deficits that were too severe to survive (modified Neurological Severity Score [mNSS] ≤ 6 or death, *n* = 9), or because of the poor quality of EEG/EMG signals, as visually inspected by experimental operators (*n* = 2). Twenty-eight rats with moderate to severe motor function deficits (mNSS > 6) on day 2 post-stroke were randomized into two groups: the FAT-C (*n* = 14) and FOR-T (*n* = 14) groups. The flowchart of the experimental design is shown in **Figure 2**.

### Evaluations

The mNSS was used to assess motor function impairments. Central fatigue was evaluated by measuring the concentrations of tryptophan (Trp) and tyrosine (Tyr) in plasma, as well as through EEG complexity. The training-induced rehabilitative effects in cortical and corticomuscular pathways were assessed using the EEG power spectral density (PSD) slope and dCMC. The EEG and EMG signals were amplified with a gain of 1750 using a neural signal acquisition system (OmniPlex Neural Signal Acquisition System, Plexon Inc., Dallas, TX, USA) with a sampling rate of 40 kHz. Recordings were taken both before and during daily training, which represented the awake and training states, respectively. EEG and EMG data were notch filtered between 49 and 51 Hz to remove artifacts and line noise, and were band-pass filtered from 2–200 Hz during the evaluation phase. Visual inspection of the EEG and EMG signals was conducted to exclude any segments or channels with motion artifacts. A total of 364 trials were used in this study.

#### Neurological function

Neurological function was graded using the mNSS on a scale of 0–18, with 0 indicating normal neurological function and 18 representing maximum functional deficits (Schaar et al., 2010; Zhang et al., 2024). The mNSS included assessments of motor function (0–6), sensory function (0–2), beam balance (0–6), and reflexes/abnormal movements (0–4). Assessments were conducted by an experimenter (who was blinded to both the training protocol and group information of the rats) on days 2 to 14, prior to the daily rehabilitation intervention.

#### Concentrations of Trp and Tyr in plasma

Central fatigue is related to 5-HT generation and reduced dopamine (Bailey et al., 1993). During exercise, free Trp and branched-chain amino acids (BCAAs) in plasma compete for a specific transporter to cross the blood–brain barrier to synthesize 5-HT, whereas Tyr is used to synthesize dopamine (Meeusen et al., 2006). Consequently, the ratio of plasma free Trp to Tyr was adopted to serve as an indicator of central fatigue, and Trp/BCAAs and Tyr/BCAAs were used to represent the concentrations of 5-HT and dopamine, respectively (Meeusen et al. 1996).

Blood samples were collected before and after training sessions to assess the central fatigue induced by FAT-C and FOR-T training. To mitigate the effects of hemostasis on health status and behavioral evaluation, blood collection was performed on day 14. After anesthesia with 5% isoflurane in oxygen (2.4 L/minute), blood was collected from the tail vein within 3 minutes to prevent recovery from central fatigue, thus ensuring that biomarker levels in the blood were not altered. Approximately (1–2) mL of blood was collected in plastic tubes containing ethylenediaminetetraacetic acid 2Na (7 mL, Solarbio, Beijing, China). The blood-collecting tubes were inverted several times to prevent hemolysis and centrifuged at 2010 × *g* for 10 minutes at 4°C to obtain plasma. The plasma samples were then stored at −80°C until analysis. For analysis, 0.1 mL of plasma sample was extracted with 0.2 mL acetonitrile and 0.3 mL 0.2 M hydrochloric acid for 1 hour with gentle agitation on a shaker at room temperature (26°C). Each sample was filtered through a 0.22 µm pore membrane filter. Next, 10 µL of sample was added to an ultra-high-performance liquid chromatography (UHPLC) vial with 70 µL borate buffer and 2 µL AccQ-Tag reagent (Waters Corporation, Milford, MA, USA). The reaction mixture was maintained at room temperature for 1 minute before being heated at 55°C for 10 minutes. After cooling, 1 µL of the mixture was analyzed using a UHPLC–Orbitrap mass spectrometry system (UHPLC system: Vanquish; mass spectrometry system: Q Exactive; Thermo Fisher Scientific, Waltham, MA, USA). Data were acquired on the Q-Exactive using Xcalibur 4.1 (Thermo Fisher Scientific), and processed using TraceFinder 4.1 Clinical (Thermo Fisher Scientific). The change rate of central fatigue related amino acids was calculated as:


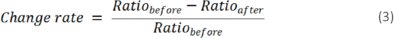


where the *Ratio*_before_ was the Trp/Tyr, Trp/BCAAs, or Tyr/BCAAs acquired from the blood collected before training, and the *Ratio*_after_ was the Trp/Tyr, Trp/BCAAs, or Tyr/BCAAs acquired from the blood collected after training.

#### Lempel–Ziv complexity in electroencephalography

Lempel–Ziv complexity (LZC) measures the degree of redundancy or randomness within a sequence. In the context of EEG signal processing, LZC is frequently applied to assess exercise-induced mental fatigue, and a reduction in LZC is interpreted as a decline in the brain’s capacity to sustain task performance (Xu et al., 2018). Consequently, we used the magnitude of the decrease in LZC within the motor cortex to characterize central fatigue originating from this region. The LZC calculates the number of new sub-sequences in one segment of the EEG signal. The detailed method has been described previously (Das and Puthankattil, 2022). The pre-processed EEG signal during treadmill training was segmented into 5-second epochs to calculate the LZC value, and the LZC drop rate was evaluated as:


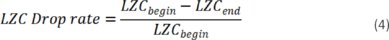


where the *LZC*_begin_ and *LZC*_end_ were the average LZC values calculated in the first and last 2 minutes, respectively, of the 30-minute treadmill training.

#### Electroencephalography power spectral density slope in the resting state

The EEG PSD slope was used to evaluate stroke-related EEG alterations in wide-band power and recovery in the AFF hemisphere. The EEG PSD slope was calculated as described previously (Lanzone et al., 2022). Briefly, pre-processed EEG data in the resting state (3 minutes) was segmented into 5-second intervals. The power spectra (2–45 Hz) of each segment were log-transformed for each frequency bin (1 Hz), and a linear curve was fitted to the log-transformed data to estimate the PSD slope of EEG. To minimize error, the mean EEG PSD slope of all segments was taken as the EEG PSD slope in the resting state.

The symmetry of the PSD slope (*PSDslopeSI*) (symmetry index [SI]) in the AFF and UN in the resting state was used to evaluated the asymmetry of the two hemispheres as follows:


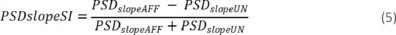


where the *PSD*_slopeAFF_ and the *PSD*_slopeUN_ represented the PSD slope value in the AFF and UN hemisphere, respectively, in the resting state.

#### Directed corticomuscular coherence

dCMC reflects the interactions between the descending and ascending pathways that run between the motor cortex and peripheral muscles. We used the dCMC to investigate interaction changes during post-stroke FOR-T and FAT-C. The dCMC was estimated using an autoregressive model as previously reported (Zhou et al., 2021). Briefly, the EEG (*X*_1_(*t*)) and EMG (*X*_2_(*t*)) data were segmented into 1-s lengths and depicted as an autoregressive model:


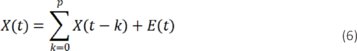


where *A*_k_ was a 2 × 2 matrix of coefficients describing the causal influence of the signals at lag *k* on the signals at lag zero, *p* represented the order of autoregressive model, and *E*(*t*) indicated the prediction errors. Equation (6) was then transformed to the frequency domain to obtain the transfer function *H*(*f*):


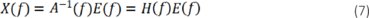


where *H*_12_(*f*) represents the transfer functions that give the influence of EMG on EEG. The directed coherence from EMG to EEG was defined as


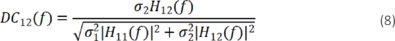


where *σ*_1_ and *σ*_2_ represented the variance of prediction errors in EEG and EMG, respectively.

The significant level was estimated using numerical Monte Carlo simulation. Briefly, two independent Gaussian random vectors with the same number of trials as the original data were generated, and the directed coherence was calculated. This procedure was repeated 50 times with different random numbers. Directed coherence estimates at all frequencies and for all simulations were rank ordered, and the 95th percentile was used as an approximate *P* < 0.05 significance level. Non-significant dCMC, with values lower than the significance level, were set to 0 for subsequent statistical comparisons (Zhou et al., 2021). The dCMC amplitude was normalized and the mean dCMCs in the first and last 2 minutes during one running trial were compared to evaluate the effects of FOR-T and FAT-C training.

### Statistical analysis

No statistical methods were used to predetermine sample sizes; however, our sample sizes were similar to those reported in previous publications (Wang et al., 2023; Zhao et al., 2024). Normality was evaluated using the Kolmogorov–Smirnov test with a significance level of 0.05. All data samples conformed to normal distributions (*P* < 0.05). To investigate the effects of time and group factors, differences in the MPF drop rates, behavior scores, LZC drop rates, EEG PSD slopes, and baseline dCMC between the FOR-T and FAT-C groups were evaluated using two-way analysis of variance (ANOVA) and post hoc Bonferroni tests (more than two groups) or Student’s *t*-test. Both the significance level and effective size (EF) were evaluated. The intragroup changes at different timepoints and intergroup differences at the same timepoint (more than two groups) were evaluated using one-way ANOVA followed by *post hoc* Bonferroni tests, to strictly control the family-wise error rate at *α* = 0.05 and minimize the risk of type I errors across multiple comparisons. Intragroup changes in free Trp/BCAAs on day 14 before and after training, changes in dCMC amplitude at the beginning and end of a single running trial in the FOR-T and FAT-C groups, and the dominant patterns of dCMC in the descending and ascending pathways were compared using paired *t*-tests. The intergroup comparison of behavioral scores at the same timepoint and the change rate of free Trp/BCAAs on day 14 before and after training were evaluated using Student’s *t*-test. *P* < 0.05 was considered significant. All statistical analyses were performed using IBM SPSS Statistics, version 20.0 (IBM Corp., Armonk, NY, USA).

## Results

### Peripheral fatigue is constrained during fatigue-controlled training training

**Figure 3:**
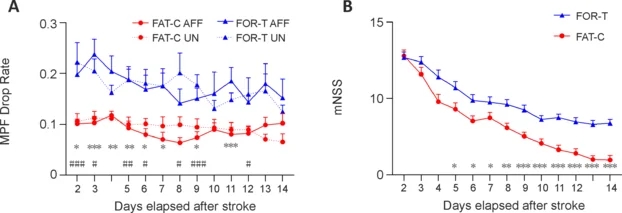
Training strategy evaluation and motor function assessment. (A) MPF drop rates of the UN and AFF hindlimbs in the FAT-C and FOR-T groups from day 2 to day 14 post-stroke (*n* = 13 per group). The “*” indicates significant differences between the MPF drop rates of the FAT-C and FOR-T groups in the AFF and UN hindlimbs, respectively. (B) mNSSs of the FAT-C and FOR-T groups from day 2 to day 14 poststroke (*n* = 13 per group). The “*” indicates significant differences between the FAT-C and FOR-T groups. Data are presented as the mean ± SEM at each timepoint. “*” and “#” indicate significant differences between the MPF drop rates of the FAT-C and FOR-T groups in the AFF and UN hindlimbs, respectively. *P < 0.05, **P < 0.01, ***P < 0.001; #*P* < 0.05, ##*P* < 0.01; ###*P* < 0.001. AFF: Affected; UN: unaffected. FAT-C: fatigue-controlled training; FOR-T: forced training; mNSS: modified Neurological Severity Score; MPF: mean power frequency.

To evaluate the peripheral FAT-C strategy, the MPF drop rates during training in the AFF and UN hindlimbs of the FAT-C and FOR-T groups were evaluated from days 2 to 14 post-stroke. Two-way ANOVA revealed that the overall MPF drop rate varied significantly with respect to the time point and group factors (two-way ANOVA, timepoint: *P* < 0.006, EF = 0.052; group: *P* < 0.001, EF = 0.294). In terms of intergroup differences on different days, the FOR-T AFF group exhibited a higher MPF drop rate than the FAT-C AFF group from days 2 to 11, excluding days 8 and 10 (one-way ANOVA for intergroup differences at the same timepoint, *P* ≤ 0.002, EF ≥ 0.282, Bonferroni’s *post hoc*: *P* ≤ 0.026, **Figure 3A**). On the UN side, the FOR-T group exhibited a higher MPF drop rate than the FAT-C UN group from days 2 to 12, except on days 4, 7, 10, and 11 (one-way ANOVA for intergroup differences at the same timepoint, *P* ≤ 0.006, EF ≥ 0.283, Bonferroni’s *post hoc*: *P* ≤ 0.035, **Figure 3A**). These findings indicate that the fatigue level was more constrained in the FAT-C group than in the FOR-T group, thus indicating the effectiveness of the FAT-C platform. This observation aligns with the results of our previous study (Xu et al., 2020) in which a similar experimental design was used for the FAT-C and FOR-T groups.

### Fatigue-controlled training strategy leads to better functional outcomes compared with forced training strategy

The overall mNSS and sub-scores in the FOR-T and FAT-C groups were assessed from days 2 to 14 post-stroke. We further calculated the sample size using the mean and standard deviation of mNSS in the FAT-C and FOR-T groups using G*power. The effect size was 11.586 and *n* = 13 was sufficient for further analysis. We note that the overall mNSS varied significantly with respect to the time point and group factors (two-way ANOVA, timepoint: *P* < 0.001, EF = 0.614, group: *P* < 0.001, EF = 0.232). Student’s *t*-test revealed significantly lower mNSS in the FAT-C group than in the FOR-T group from days 4 to 14 (*P* ≤ 0.035, **Figure 3B**), indicating better motor function recovery in the FAT-C group. In an intra-group comparison of rehabilitation speed, the FAT-C group exhibited a significant reduction in mNSS from day 4 compared with baseline, whereas a significant reduction compared with baseline was observed from day 5 in the FOR-T group (one-way ANOVA for intragroup changes at different timepoints, FAT-C: *P* < 0.001, EF = 0.680, Bonferroni’s *post hoc* tests *P* ≤ 0.002; FOR-T, *P* = 0.000, EF = 0.532, Bonferroni’s *post hoc* tests *P* ≤ 0.003, **Figure 3B**), which suggests a faster recovery period in the FAT-C group. Although both the FOR-T and FAT-C groups exhibited motor function recovery, better motor function recovery was observed in the FAT-C group (**Figure 3B**).

Furthermore, the detailed motor sub-score and beam balance sub-score in the FOR-T and FAT-C groups were assessed from days 2 to 14 post-stroke to identify the main factors involved in motor function recovery. Two-way ANOVA revealed that the motor and beam balance sub-scores varied significantly with respect to the time point and group factors (two-way ANOVA, motor sub-score: timepoint: *P* < 0.001, EF = 0.417, group: *P* < 0.001, EF = 0.073; beam balance sub-score: timepoint: *P* < 0. 001, EF = 0.531, group: *P* < 0.001, EF = 0.240). The FAT-C group exhibited a significantly better motor sub-score than the FOR-T group on days 4, 8, and 10 to 14 (Student’s *t*-test, *P* ≤ 0.029, **Additional Figure 1**). The FAT-C group displayed a significantly better beam balance sub-score than the FOR-T group from days 5 to 14 (except day 7, Student’s *t*-test *P* ≤ 0.030, **Additional Figure 1**). The inter-group differences were more concentrated in the beam balance sub-score than in the motor sub-score. These results indicate both faster and superior motor function recovery—especially, in movement and balance ability—with individualized FAT-C than with FOR-T. Further analysis suggested that the superior recovery was mainly observed in the beam balance sub-score, which suggests improved coordination and balance ability (Luong et al., 2011) in the FAT-C group compared with the FOR-T group. These results further emphasize the importance of an individualized training strategy in early stroke rehabilitation.

### Central fatigue is constrained in the fatigue-controlled training group

During prolonged physical training, free Trp and Tyr in plasma compete with BCAAs to traverse the blood–brain barrier, thereby leading to the synthesis of 5-HT and dopamine. This results in a feeling of lethargy and a decline in neural drive, ultimately leading to central nervous system fatigue (Meeusen and Watson, 2007; Soares et al., 2007). Because the ratios of free Trp/BCAAs and Tyr/BCAAs are in alignment with the accumulation of 5-HT and dopamine in the brain (Hasegawa et al., 2008; Teixeira-Coelho et al., 2014; Cordeiro et al., 2017), changes in free Trp/Tyr before and after training in the FAT-C and FOR-T groups were assessed to evaluate central fatigue.

**Figure 4:**
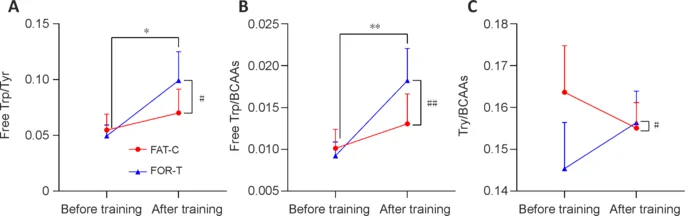
Central fatigue based on amino acids in the FOR-T and FAT-C groups. (A–C) The values of free Trp/Tyr (A), free Trp/BCAAs (B), and Tyr/BCAAs (C) before training and after training in the FAT-C and FOR-T groups on day 14 (*n* = 13 per group). Data are presented as the mean ± SEM. *P < 0.05, **P < 0.01, before *vs*. after training; #*P* < 0.05, ##*P* < 0.01, FAT-C *vs*. FOR-T group (Student’s *t-*test). BCAAs: Branched-chain amino acids; FAT-C: fatigue-controlled training; FOR-T: forced training; Trp: tryptophan; Tyr: tyrosine.

A significantly higher value of free Trp/Tyr was observed after training compared with before training in the FOR-T group, whereas no significant change was identified in the FAT-C group (FOR-T: *P* = 0.018, FAT-C: *P* = 0.103, **Figure 4A**). The FOR-T group also exhibited a higher change rate of free Trp/Tyr than the FAT-C group (*P* = 0.040, **Figure 4A**). These results suggest that central fatigue generation only occurs in the FOR-T group.

In addition, Trp/BCAAs and Tyr/BCAAs were analyzed to investigate the concentration of 5-HT and dopamine in brain tissue. A significantly higher value of free Trp/BCAAs was identified after training compared with before training in the FOR-T group, whereas no significant change was identified in the FAT-C group (FOR-T: *P* = 0.002, FAT-C: *P* = 0.189, **Figure 4B**). For the intergroup comparison, the FOR-T group exhibited a greater change rate of free Trp/BCAAs than the FAT-C group (*P* = 0.002, **Figure 4B**). For changes of Tyr, no significant differences were noted between after training and before training in the FAT-C and FOR-T groups (FAT-C: *P* = 0.132, FOR-T: *P* = 0.375, **Figure 4C**). However, the change rate of Tyr/BCAAs was significantly different between the FAT-C and FOR-T groups (*P* = 0.018, **Figure 4C**). These results indicate that central fatigue–related neurotransmitters are generated during FOR-T, and suggest that this change is mainly attributable to increased 5-HT, whereas FAT-C training constrains 5-HT synthesis. The intense release of 5-HT may reduce motoneuron activity and exacerbate central fatigue during prolonged sustained contractions (Kavanagh et al., 2019). Furthermore, 5-HT is related to plasticity injury, which may constrain the recovery of the AFF hemisphere, leading to further imbalances between the two hemispheres. Additionally, exercise-induced fatigue impairs both corticostriatal long-term potentiation and long-term depression (Ma et al., 2018), and these are essential for motor function rehabilitation. We therefore propose that individualized training might constrain central fatigue by constraining the peripheral fatigue level, whereas in the FOR-T group, central fatigue–related motor drive reduction and plasticity injury may have led to poorer motor function recovery.

**Figure 5:**
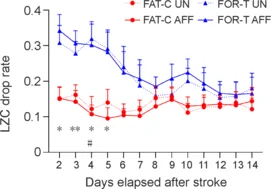
Central fatigue in the motor cortex of the FOR-T and FAT-C groups. LZC drop rates of the AFF/UN hemisphere in the FOR-T and FAT-C groups from day 2 to day 14 post-stroke (*n*=13 per group). Data are presented as the mean ± SEM. *P < 0.05, **P < 0.01, FOR-C AFF group *vs*. FOR-T AFF group; #*P* < 0.05, FOR-C UN group *vs*. FOR-T UN group. AFF: Affected; FAT-C: fatigue-controlled training; FOR-T: forced training; LZC: Lempel-Ziv complexity; UN: unaffected.

The LZC drop rates of the AFF/UN hemisphere in the FOR-T and FAT-C groups from days 2 to 14 post-stroke were investigated to reveal central fatigue at the motor cortex level. Only group factor varied in LZC drop rates (two-way ANOVA, group: *P* < 0.001, EF = 0.094; time: *P* = 0.841, EF = 0.018). In the AFF hemisphere, higher LZC drop rates were identified in the FOR-T group than in the FAT-C group from days 2 to 5 (one-way ANOVA for intergroup differences at the same timepoint, *P* ≤ 0.005, EF ≥ 0.259, Bonferroni *post hoc*: *P* ≤ 0.030, **Figure 5**). For the UN hemisphere, significantly higher LZC drop rates were observed in the FOR-T group than in the FAT-C group on day 4 (one-way ANOVA for intergroup differences at the same timepoint, *P* = 0.001, EF = 0.311, Bonferroni *post hoc*: *P* = 0.017, **Figure 5**). These results suggest that, at the motor cortex level, more central fatigue occurs with FOR-T.

In the late rehabilitation stage (day 14 post-stroke, around 3 month post-stroke in the clinic), although no significant difference was observed in motor drive output (as quantified by EEG complexity), the levels of central fatigue–related amino acids in plasma indicated greater central fatigue in the FOR-T group than in the FAT-C group. These findings suggest that peripheral FAT-C can also mitigate central fatigue in the late rehabilitation stage and underscore the importance of individualized fatigue monitoring–based training during the late stages of rehabilitation.

### Forced training aggravates the asymmetry of the two hemispheres by strengthening the unaffected hemisphere

**Figure 6:**
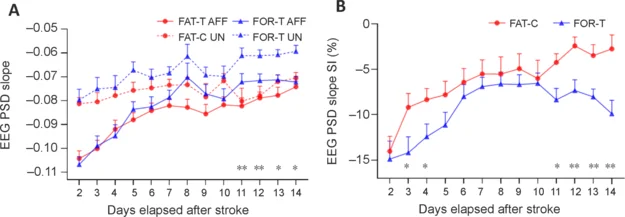
The EEG PSD slope and its SI in the FOR-T and FAT-C groups from day 2 to day 14 poststroke. (A) The EEG PSD slope in the AFF/UN hemispheres of the FOR-T and FAT-C groups from day 2 to day 14 poststroke (*n* = 13 per group). *P < 0.05, **P < 0.01, FAT-C UN *vs.* FOR-T UN. (B) The EEG PSD slope SI in the FOR-T and FAT-C groups from day 2 to day 14 poststroke (*n* = 13 per group). *P < 0.05, **P < 0.01, FAT-C *vs.* FOR-T. Data are presented as the mean ± SEM. AFF: Affected; EEG: electroencephalography; FAT-C: fatigue-controlled training; FOR-T: forced training; PSD: power spectral density; SI: symmetry index; UN: unaffected.

The EEG PSD slopes of the UN/AFF hemispheres in the FAT-C and the FOR-T groups from days 2 to 14 post-stroke were investigated to evaluate the effects of rehabilitation on the activity of each hemisphere. An EEG PSD slope reduction in the AFF side revealed the stroke-induced injury and reflected the increased power in lower frequency bands and decreased power in higher frequency bands. Two-way ANOVA suggested that the overall EEG PSD slope varied significantly with respect to the time point and group factors (two-way ANOVA, timepoint: *P* < 0.001, EF = 0.246; group: *P* < 0.001, EF = 0.248). In the late stage of rehabilitation, significantly stronger EEG PSD slopes were noted in the FOR-T UN group than in the FAT-C UN group from days 11 to 14 (one-way ANOVAs for intergroup differences at the same timepoint, *P* ≤ 0.002, EF ≤ 0.298, Bonferroni’s *post hoc*: *P* ≤ 0.023, **Figure 6A**). These results indicate that the FOR-T strategy may help to strengthen the UN side, which then constrains recovery on the AFF side.

The SI values of the EEG PSD slopes of the FAT-C and FOR-T groups from days 2 to 14 post-stroke were used to evaluate inter-hemispheric balance. Significantly higher SI values were observed in the FAT-C group than in the FOR-T group on days 3, 4, and 11 to 14 (Student’s *t-*test, *P* ≤ 0.043, **Figure 6B**). These results indicate that the greater inter-hemispheric imbalance in the FOR-T group may mainly be related to the strength of the UN side. This imbalance may further constrain motor function recovery.

### Fatigue reduces drive from the motor cortex to the target muscle

The normalized descending and ascending dCMC in the early and late stages of a single treadmill training trial from days 2 to 14 were evaluated to investigate the influence of fatigue training on corticomuscular pathways.

**Figure 7:**
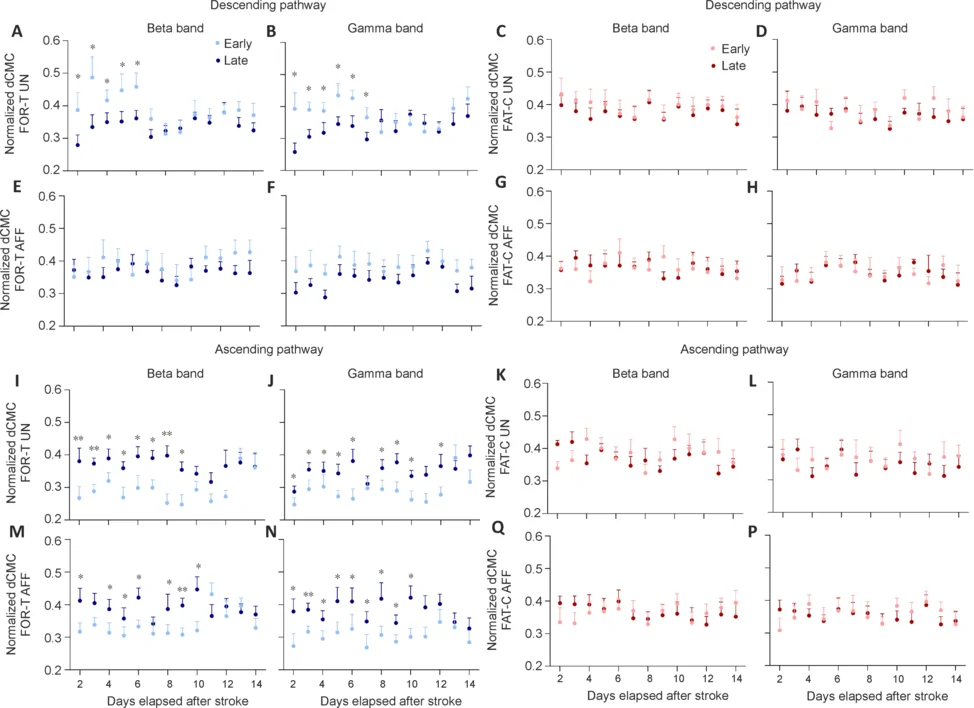
The normalized dCMC in the early and late stages of running from day 2 to day 14 poststroke in the FOR-T and FAT-C groups. (A–D) Normalized descending dCMC in the beta and gamma bands of the FOR-T and FAT-C UN groups. (E-H) The normalized descending dCMC in the beta and gamma bands of the FOR-T and FAT-C AFF groups. (I–L) The normalized ascending dCMC in the beta and gamma bands of the FOR-T and FAT-C UN groups. (M–P) The normalized ascending dCMC in the beta and gamma bands of the FOR-T and FAT-C AFF groups (*n* = 13 per group). *P < 0.05, **P < 0.01, early stage *vs.* late stage. Data are presented as the mean ± SEM. AFF: Affected; dCMC: directed corticomuscular coherence; FAT-C: fatigue-controlled training; FOR-T: forced training; UN: unaffected.

In the descending pathway, both the beta and gamma band dCMC were significantly lower in the late stage than in the early stage in the FOR-T UN group (paired *t*-test, beta band, days 2 to 6, *P* ≤ 0.0344, **Figure 7A**; gamma band, days 2 to 7, *P* ≤ 0.049, **Figure 7B**), whereas no significant differences between the early and late stages were identified in the FOR-T AFF, FAT-C UN, and FAT-C AFF groups (paired *t*-test, *P* > 0.050, **Figure 7C** and **D**). In the AFF side, no significant differences between the early and late stages were observed in the FOR-T or FAT-C groups (paired *t*-test, *P* > 0.050, **Figure 7E–H**). These results suggest that the motor cortex may decrease the descending motor drive in response to an extreme fatigue state, to maintain a dynamic homeostasis state and avoid injury.

In the ascending pathway, both beta and gamma band dCMC exhibited significant reductions in the late stage compared with the early stage in the FOR-T UN and FOR-T AFF groups (paired *t*-test, beta band, FOR-T UN, days 2 to 9, *P* ≤ 0.048, **Figure 7I**; gamma band, FOR-T UN, days 2 to 12 except days 7 and 11, *P* ≤ 0.049, **Figure 7J**; beta band, FOR-T AFF, days 2 to 10 except days 3 and 7, *P* ≤ 0.044, **Figure 7M**; gamma band, FOR-T AFF, days 2 to 10, *P* ≤ 0.048, **Figure 7N**). No significant differences between the early and late stages were identified in the FAT-C UN or FAT-C AFF groups (paired *t*-test, *P* > 0.05; **Figure 7K**, **L**, **O**, and **P**). These results suggest that, in the extreme fatigue state, in which the motor cortex decreases drive to the muscle, muscle may increase the ascending feedback to compensate, thus allowing stable movement.

### Directed corticomuscular coherence dominant pattern during rehabilitation

**Figure 8:**
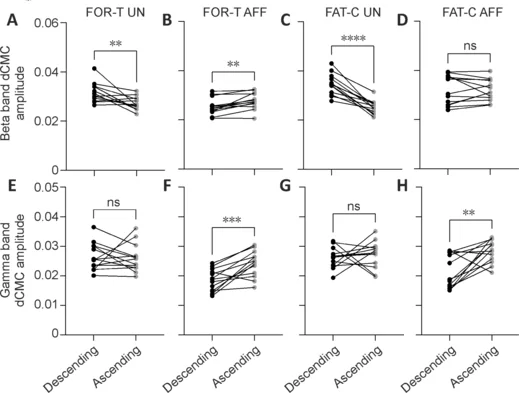
Beta- and gamma-band dCMC-dominant patterns in the FOR-T and FAT-C groups. The beta band dCMC dominant main effect in the FOR-T UN group (A), FOR-T AFF group (B), FAT-C UN group (C), FAT-C AFF group (D), and gamma band dCMC dominant main effect in the FOR-T UN group (E), FOR-T AFF group (F), FAT-C UN group (G), and FAT-C AFF group (H) (*n* = 13 per group). **P < 0.01, ***P < 0.001, ****P < 0.0001, descending *vs.* ascending dCMC. Data are presented as the mean ± SEM. AFF: Affected; dCMC: directed corticomuscular coherence; FAT-C: fatigue-controlled training; FOR-T: forced training; ns: not significant; UN: unaffected.

The dCMC dominant main effect in the beta band is shown in **Figure 8A–D**. For the UN side, the descending dCMC was significantly higher than the ascending dCMC in both the FAT-C UN and FOR-T UN groups (paired *t*-test, FAT-C UN group: *P* < 0.0001; FOR-T UN group: *P* =0.005, **Figure 8A** and **C**). On the AFF side, there was no significant difference between the descending and ascending dCMC in the FAT-C group (paired *t*-test, *P* > 0.05, **Figure 8D**), whereas a significantly higher ascending dCMC was noted compared with the descending dCMC in the FOR-T group (paired *t*-test, *P* = 0.002, **Figure 8B**). These results suggest that stroke disrupts the descending dominant main effect in the beta band. In the FOR-T group, training led to a reduction in descending dCMC and an increase in ascending dCMC. The control of muscles by the single-synaptic corticospinal tract from the cortex was impaired, thus significantly weakening the descending pathway. As a result, the group was unable to achieve descending dominant movement control; instead, they exhibited an ascending dominance phenomenon that further amplified the dominant pattern difference, which was reflected as the ascending dominant main effect. By contrast, for the FAT-C AFF group, no significant dominant main effect was observed, suggesting a recovery in descending control ability.

The dCMC dominant main effect in the gamma band is shown in **Figure 8E–H**. On the AFF side, the ascending dCMC was significantly higher than the descending dCMC in both the FAT-C AFF and FOR-T AFF groups (paired *t*-test, FAT-C AFF group: *P* = 0.004, FOR-T AFF group: *P* = 0.0007, **Figure 8F** and **H**). For the UN side, no significant differences were noted between the descending and ascending dCMC in both the FAT-C UN and FOR-T UN groups (paired *t*-test, no dominant pattern, FAT-C UN group: *P* = 0.555, FOR-T UN group: *P* = 0.774, **Figure 8E** and **G**). These results suggest that there are no differences between the FAT-C and FOR-T groups in terms of the gamma band dominant main effect.

## Discussion

The low rehabilitation efficiency of post-stroke motor function rehabilitation remains an important challenge. Our study aimed to investigate how individualized training benefits post-stroke motor function rehabilitation and to offer potential solutions for high-efficiency rehabilitation. In the present study, we compared the rehabilitative effects of individualized FAT-C and FOR-T in a rat model of stroke. We demonstrated that an individualized peripheral FAT-C strategy improved post-stroke motor function recovery by limiting central fatigue, achieving better inter-hemispheric balance, and enhancing descending movement control. This is the first study to explore how individualized peripheral FAT-C improves rehabilitation outcomes in a stroke model, thus providing a promising approach for enhancing post-stroke rehabilitation efficiency.

To further investigate the rehabilitative effects of individualized training, we conducted analyses at the cortical and corticomuscular pathway levels. At the cortical level, the rehabilitative effects were related to inter-hemispheric balance, which is disrupted by stroke because of reduced inhibitory projections from the AFF hemisphere to the UN hemisphere (Chen et al., 2023; Mauro et al., 2024). In the current study, stroke disrupted the inter-hemispheric symmetry of the EEG PSD slope on day 2, and a significantly better symmetry index was observed in the FAT-C group during the late stage of rehabilitation. This finding suggests that, compared with the FOR-T strategy, FAT-C can alleviate stroke-induced inter-hemispheric imbalance, bringing it to a more balanced state. The observed difference in inter-hemispheric imbalance may be related to the varying fatigue levels caused by different training strategies.

Peripheral fatigue and central fatigue maintain a dynamic homeostasis through negative feedback (Tornero-Aguilera et al., 2022; Myers, 2024). As peripheral fatigue increases during intensive training, it generates central fatigue, thus further constraining motor cortex output. This concept aligns with the significantly higher peripheral and central fatigue that was observed in the FOR-T group, as evaluated by LZC drop rate and plasma Trp and Tyr, respectively. A reduction in EEG complexity is considered a potential indicator of decreased motor drive commands, which may be attributed to central fatigue (Ibáñez-Molina et al., 2018; Xu et al., 2018). This reduction in motor drive is consistent with findings of exhaustion in tasks such as pull-ups (Tergau et al., 2000) and thumb isometric contractions (Kotan et al., 2015). By contrast, by dynamically adjusting the training intensity based on peripheral fatigue levels, the FAT-C method effectively limited the development of central fatigue, thus preserving motor cortex excitability and supporting cortical neuroplasticity, which is essential for recovery. The dynamic homeostasis between peripheral and central fatigue underscores the importance of fatigue modulation in rehabilitation. Our results indicate that controlling peripheral fatigue not only prevents physical overexertion but also mitigates central fatigue, thereby facilitating better motor function outcomes. These findings suggest that rehabilitation strategies should prioritize fatigue monitoring, thus offering a pathway to more efficient and personalized post-stroke interventions.

In corticalmuscular pathway, the FOR-T group experienced a reduction in descending dCMC and an increase in ascending dCMC. A decline in descending dCMC indicates a reduction in the ability of the motor cortex to control the muscles and a decoupling of signals between peripheral muscles and the motor cortex, which may be linked to the attenuation of external input to motor neurons caused by suppression of the motor cortex by muscle afference in group III/IV in response to a peripheral fatigue state (Taylor et al., 2016). In a previous study, we demonstrated that FOR-T under extreme fatigue levels can potentially exacerbate damage to the AFF striatum (Xu et al., 2020). This damage further reduces the inhibitory projections from the AFF hemisphere, leading to increased excitability in the UN hemisphere; this disrupts inter-hemispheric balance and constrains the recovery of the AFF hemisphere. Together, these findings suggest that overloaded training intensity exacerbates stroke-induced inter-hemispheric asymmetry, thereby further hindering recovery in the AFF hemisphere in the FOR-T group. The healthy motor cortex may increase excitability during the fatigue state as a compensatory role to maintain motor output, whereas stroke reduces cortical excitability and increases excitability thresholds to stimulation in the AFF motor cortex (Kuppuswamy et al., 2015; Hogan et al., 2020; Hsu et al., 2023), making it difficult for the AFF hemisphere to increase motor drive.

The present study also highlighted the critical role of inter-hemispheric balance in early-stage rehabilitation, and how FAT-C may facilitate this rebalancing. Stroke disrupts inter-hemispheric balance, leading to reduced excitability in the AFF hemisphere. This imbalance in motor activation is associated with motor deficits (Graterol Pérez et al., 2022; Tam et al., 2024), which can recover with motor restoration (Tang et al., 2015). In our study, the FOR-T group exhibited poor inter-hemispheric balance, with stronger activation in the UN hindlimb. Overloaded training led to the excessive use of both limbs to accomplish movements, which caused stress and injury, and may result in reduced plasticity (Sun et al., 2014). In clinical research, superior motor recovery was observed in the UN cortex suppression group compared to AFF cortex activation group (Wang et al., 2020b), thereby emphasizing the importance of suppressing the UN hemisphere. For clinical stroke rehabilitation, if structural reserve is high, the interhemispheric balance model can predict recovery better than the compensation model, whereas with low structural reserve, UN compensation may dominate (Li et al., 2022). Thus, structural measurements may be necessary to further enhance the efficiency of the FAT-C strategy in clinical applications.

Voluntary movement execution requires the coordination of descending motor drive and ascending sensory feedback. An increase in ascending dCMC is associated with the instability of motor control caused by excessive fatigue. Movement execution relies on the constant comparison of ascending sensory feedback with expected feedback to complete motion calibration. However, when muscles are fatigued, the control ability of the motor cortex weakens, thus impairing its response to ascending feedback. This disruption makes it difficult to appropriately respond to feedback and challenges effective motion calibration. As a result, the ascending pathway is continuously enhanced. Similar phenomena have been observed in a previous study in which subjects compensated for unstable muscle states by increasing ascending dCMC to maintain exercise output (Zhou et al., 2021). However, in the later stage of the rehabilitation training intervention (days 10 to 14), the enhancement of the ascending pathway was reduced, indicating that with the improvement of motor function and fatigue tolerance, exercise output during the latter phase of single running training tends to stabilize. In the present study, we observed distinct dominant patterns in the beta and gamma bands of dCMC. Previous studies have reported that the beta band primarily transmits downward motion control information (Gwin et al., 2011), whereas the gamma band mainly transmits upward sensory feedback (Liang et al., 2021). In a normal/undamaged movement state, by comparing the expected and actual incoming proprioceptive feedback, motor output instructions are constantly adjusted to complete and stabilize the motor task, showing the phenomenon of descending dominance (Baker, 2007). In the current study, the FOR-T group exhibited significantly less descending dCMC, as indicated by the ascending dominant effect in the beta band, whereas the FAT-C group did not. This finding suggests that FAT-C effectively enhances the descending pathway on the AFF side, thereby improving the ability to control the AFF hindlimb and preventing the ascending dominant pattern in beta band dCMC. As a result, the FAT-C group experienced better motor function recovery.

In the present study, we revealed differences in the dominant patterns exhibited by the beta and gamma frequency bands, which may be related to the functional differences of these bands across different paradigms. Clinical studies have revealed that during a lower limb dorsiflexion task, healthy subjects exhibit significantly higher beta band information flow in the descending direction compared with the ascending direction, whereas in the gamma band, ascending information flow is notably higher than descending flow. This suggests that in the dorsiflexion task, the beta band primarily transmits downward motor control information, whereas the gamma band mainly conveys upward sensory feedback information (Liang et al., 2021). Combining these previous results with the findings of the present study, we propose that in the stroke treadmill training paradigm in rats, both beta and gamma bands play roles in motor control and sensory feedback, collectively contributing to movement execution. However, the function of the beta band appears to be more inclined toward descending motor control output, whereas the gamma band may be more oriented toward the processing of sensory feedback.

It has been suggested that cortical involvement is unnecessary for lower limb movement in rodents because repetitive basal movements of lower mammals are primarily regulated by subcortical and spinal cord networks (Stuart and Hultborn, 2008). However, recent research has revealed the consistent activation of motor neurons in the lower extremity motor cortex and the hindlimbs of rats during ground walking. Additionally, differences in motor cortex activation have been observed across walking paradigms with varying levels of participation and difficulty (Li et al., 2021). In the present study, we further demonstrated that cortical participation is required for rats to perform lower extremity running after a stroke.

Early rehabilitation interventions (acute/subacute periods) offer the potential to leverage the critical window of neural plasticity, thus leading to substantial improvements in behavioral and functional outcomes. Overloaded training can activate the sympathetic–adrenal–medullary and hypothalamus–pituitary–adrenal axes, leading to the release of stress and catabolic hormones as well as inflammatory cytokines (Sun et al., 2014; Reddin et al., 2022), which may hinder functional recovery. We found that the FOR-T group reached a plateau in the mNSS score, whereas the FAT-C group continued to show a decreasing trend at the end of the rehabilitation period. This observation suggests that the FOR-T strategy may restrict the upper limit of motor function recovery. In contrast, the individualized FAT-C approach could facilitate further improvement in rehabilitation outcomes over the long term. In clinical practice, the implementation of individualized and moderate training strategies is often constrained by limited medical resources and personnel, which presents challenges for tailoring rehabilitation plans to meet the specific needs of each patient. The peripheral FAT-C strategy used in the present study represents a potential solution for achieving safe, early rehabilitation interventions with high efficiency in clinical settings. This approach visualizes an individual’s specific muscle fatigue level and uses feedback to adjust the training strategy. In clinical practice, the EMG signal—recorded from the target muscle with non-invasive EMG electrodes—can be applied for peripheral fatigue monitoring and modulation. By tailoring the rehabilitation protocol in this way, the risks associated with overtraining are minimized and the overall efficiency of the rehabilitation process is enhanced, even with limited medical resources.

## Limitations

The current study demonstrated the rehabilitative effects of a peripheral fatigue–controlled rehabilitation strategy using a rat model of stroke. However, there are some limitations, and future work should be discussed. First, we only evaluated rehabilitation outcomes using behavioral scores, without any measurements of injury volume, and further analysis is necessary to compare the differences in injury volume between groups. Second, peripheral fatigue levels were monitored solely through EMG signals. Future studies should incorporate additional biomarkers, such as heart rate or percentage of maximal oxygen uptake, to assess general exercise-induced fatigue rather than focusing on a single muscle group (Li, 2022); this might provide more general and stable information to evaluate the fatigue state. Third, the fatigue threshold was fixed during rehabilitation training. It would be valuable to explore whether the use of other fatigue thresholds or modulating fatigue tolerance during rehabilitation can lead to better behavioral outcomes. An adaptive fatigue threshold might further enhance rehabilitation efficiency because, as motor function improves, training intensity tolerance tends to increase (Fabero-Garrido et al., 2022), and a previous study reported that gradually increasing intensity may benefit motor function recovery. A dynamic threshold may therefore be better; however, further investigation is required to clarify how to determine the dynamic threshold. Lastly, central fatigue was evaluated through plasma analysis on day 14 only. It is not clear whether this single time point sufficiently represents the trends in central fatigue from days 2 to 14, because another central fatigue biomarker—LZC—was not consistent from days 2 to 14. For future research, longitudinal monitoring of plasma will be necessary to provide a more comprehensive understanding of its progression.

## Conclusion

The present study revealed that individualized peripheral FAT-C effectively mitigated central fatigue. This approach promoted inter-hemispheric rebalancing and the recovery of motor drive output, thus facilitating rehabilitation of the AFF hemisphere rather than over-strengthening the UN hemisphere, as observed with overloaded FOR-T. Our findings provide further insights into how individualized training strategies may benefit post-stroke motor function recovery and offer a potential solution for achieving high-efficiency post-stroke motor function rehabilitation.

## Additional file:

***Additional Figure 1:*** *Subscore evaluation in the FAT-C and FOR-T groups.*

Additional Figure 1 Subscore evaluation in the FAT-C and FOR-T groups.
(A, B) Motor (A) and beam balance (B) subscores of the FAT-C and FOR-T groups from day 2 to day 14 poststroke (*n* = 13 per group). FAT-C group *vs*. FOR-T group. ^*^P < 0.05, ^**^P < 0.01, ^***^P < 0.001. Data are presented as the mean ± SEM. FAT-C: fatigue-controlled training; FOR-T: forced training.

## Data availability statement:

*All relevant data are within the paper and its Additional files.*
